# Giant Enhancement of Radiative Recombination in Perovskite Light-Emitting Diodes with Plasmonic Core-Shell Nanoparticles

**DOI:** 10.3390/nano11010045

**Published:** 2020-12-27

**Authors:** Mikhail A. Masharin, Alexander S. Berestennikov, Daniele Barettin, Pavel M. Voroshilov, Konstantin S. Ladutenko, Aldo Di Carlo, Sergey V. Makarov

**Affiliations:** 1 Department of Physics and Engineering, ITMO University, 197101 St. Petersburg, Russia; mikhail.masharin@metalab.ifmo.ru (M.A.M.); a.berestennikov@metalab.ifmo.ru (A.S.B.); k.ladutenko@metalab.ifmo.ru (K.S.L.); s.makarov@metalab.ifmo.ru (S.V.M.); 2 Centre for Hybrid and Organic Solar Energy (CHOSE), Department of Electronic Engineering, University of Rome Tor Vergata, 00133 Rome, Italy; aldo.dicarlo@uniroma2.it; 3 Department of Electronic Engineering, Universita Niccoló Cusano, 00133 Rome, Italy; daniele.barettin@unicusano.it

**Keywords:** halide perovskites, light-emitting diodes, core-shell nanoparticles, metal-oxide semiconductor, efficiency enhancement, drift-diffusion modeling

## Abstract

The integration of nanoparticles (NPs) into functional materials is a powerful tool for the smart engineering of their physical properties. If properly designed and optimized, NPs possess unique optical, electrical, quantum, and other effects that will improve the efficiency of optoelectronic devices. Here, we propose a novel approach for the enhancement of perovskite light-emitting diodes (PeLEDs) based on electronic band structure deformation by core-shell NPs forming a metal-oxide-semiconductor (MOS) structure with an Au core and SiO${}_{2}$ shell located in the perovskite layer. The presence of the MOS interface enables favorable charge distribution in the active layer through the formation of hole transporting channels. For the PeLED design, we consider integration of the core-shell NPs in the realistic numerical model. Using our verified model, we show that, compared with the bare structure, the incorporation of NPs increases the radiative recombination rate of PeLED by several orders of magnitude. It is intended that this study will open new perspectives for further efficiency enhancement of perovskite-based optoelectronic devices with NPs.

## 1. Introduction

In recent years, halide perovskites have attracted considerable attention from research groups worldwide due to properties [1,2,3,4,5] such as reconfigurable optical bandgap, high quantum yield of photoluminescence, large carrier mobility and lifetime, low-cost fabrication, high absorption coefficient, color purity and tunability. These remarkable properties make them superior candidates for various photonic, photovoltaic, and optoelectronic applications [6,7,8]. As a result of intensive work, the record efficiency of single-junction perovskite solar cells (SCs) exceeds 25% [9]. Significant progress has also been made in the field of PeLEDs, where the external quantum efficiency (EQE) has increased by more than 20% [10,11]. Also, the unique properties of perovskites allow both functionalities to be combined in a single device known as a light-emitting solar cell (LESC). This device performs well, both in photovoltaic and electroluminescent operation modes expanding possible applications [12,13,14,15].

Despite impressive achievements during the last decade, further efforts are needed before perovskite-based light-emitting devices will be attractive for industry. The current EQE of PeLEDs is still much lower than that of III–V and organic counterparts. Many research groups are therefore trying to overcome current limitations and drawbacks of the latest perovskite optoelectronic devices. The EQE of PeLEDs is determined by the so-called light outcoupling efficiency and the internal quantum efficiency (IQE), where the IQE is determined by the photoluminescence quantum yield, the charge balance, and the radiative efficiency.

Apart from material and structure optimization, different approaches are widely explored to improve the quality and performance of PeLEDs, including recent advances in nanophotonics [16,17,18]. For example, surface patterning leads to enhanced light outcoupling and photon recycling [19,20,21,22,23] which is beneficial for PeLEDs. Other approaches have been based on the integration of metal nanoparticles (NPs) within active or transport layers exploiting different optical and electrical effects [17,24,25]. However, the origin of the improvement reported in many of these experimental papers is not always clear due to the complex physics of coupled processes that also require thorough theoretical analysis. Therefore, a proper design of the device followed by comprehensive optimization of a numerical model would help to accelerate progress towards the superior efficiency of PeLEDs.

In this paper, we propose a novel approach for PeLED efficiency enhancement based on electronic band structure engineering with core-shell Au-SiO${}_{2}$ NPs. We optimize the morphology of core-shells and numerically study the electrical effects accompanying integration of NPs into a FAPbBr${}_{2}$I-based LESC device with transport layers developed elsewhere [15]. We demonstrate that NPs can improve the radiative recombination rate in the LESC by several orders of magnitude due to the favorable formation of metal-oxide-semiconductor (MOS) interfaces. The proposed approach paves the way for further efficiency enhancement by comprehensive optimization of perovskite-based optoelectronic devices with NPs.

## 2. Materials and Methods

The correct numerical simulation of perovskite devices is challenging since the results of the simulation are strongly dependent on the accuracy of specified material parameters (band levels, mobility of charge carriers, recombination constants, etc.) which can be uncertain to some extent. The consistency of experimental I–V curves of one device with numerically obtained results for a certain set of material parameters is not sufficient for numerical model validation [26,27]. Here, we use an approach based on a thorough fitting of the I–V curves simultaneously for two working regimes (LED and SC) and for different values of proven parameters, for example, thickness of the perovskite layer. This allows extraction of verified material parameters for our LESC devices.

### 2.1. Experimental

In order to do verification of our model, we fabricated the following dual-functional LESC architecture: Glass/ITO (200 nm)/PEDOT:PSS (30 nm)/FAPbBr${}_{2}$I/C${}_{60}$ (30 nm)/LiF (1 nm)/Ag (60 nm). First, ITO covered glass substrates were patterned with the direct lithography method. In this method, we deposited photoresist onto ITO substrate, exposed it through the mask under UV illumination for 10 min, and developed in 0.5% KOH solvent in the ultrasonic bath for 3 min. Next, we etched uncovered ITO by HCl vapor at 70° for 10 min. Patterned substrates were cleaned in an ultrasonic bath consistently for 5 min in a KOH solution, 5 min in DI-water, 5 min in acetone, 5 min in isopropanol. Then, substrates were dried at 120° for 5 min and placed for 20 min under UV ozone treatment. After that, we prepared solutions for perovskite films (FAPbBr${}_{2}$I). Precursor formamidinium iodide (FAI) with a purity of 99.5% from Sigma Aldrich was dissolved in a mixture of 99.5% of waterless Dimethylformamide (DMF) and Dimethyl sulfoxide (DMSO) solvent in relation 7:3 molarity 0.8 moles. After FAI completely dissolved, we added the obtained solution into Lead (II) bromide (PbBr${}_{2}$) with a purity of 99.999%. The resulting solution was stirred at 200 rpm and 70° for 24 h.

Patterned and cleaned ITO substrate was covered with PEDOT:PSS by spin-coating method. Solution of PEDOT:PSS was deposited on the substrate in the air and then accelerated with 800 rpm${}^{2}$ to 3000 rpm for 1 min. In this process, the solution uniformly distributed on the substrate and dried fast, forming a gel-like thin film. After this, we annealed film at 150° for 10 min.

Perovskite films were synthesized on the PEDOS:PSS layer in a glove box in a dry nitrogen atmosphere with a two-step spin-coating method. First, we drop on the substrate 150 μL of the perovskite solution for spin-coating. In the first stage, spin-coater started to spin the substrate at 500 rpm for 20 s with an acceleration 100 rpm${}^{2}$. The solution was uniformly spread on the substrate and started to dry. In the second stage, substrate was accelerated with 500 rpm${}^{2}$ up to 3000 rpm for 15 s and the process of fast drying begun. Solution on the substrate became oversaturated and 8 s before the end of the rotation, we dripped 1100 μL of antisolvent (diethyl ether), which washed away solvent remains and perovskite film instantly formed. After this, we preheated films at 60° for 5 min and then annealed them at 100° for 10 min.

Electron transport layer (fullerene C${}_{60}$), hole blocking layer (LiF) and silver contact were deposited in the evaporator vacuum chamber. First, we fixed substrates with PEDOT:PSS and perovskite on the holder inside and put the masks on them. Then, we pumped out the atmosphere in the chamber to 10${}^{-4}$ Pa and started to heat the material (C${}_{60}$ or silver). Under the heat, the material transforms into vapor and spreads up. When the vapor achieves the substrates it uniformly condensates. To control this process, quartz resonators are used as a sensor. By changing the eigenfrequencies of the resonator it is possible to calculate the thickness of deposited material. There are several issues in this process, connected with the type of materials or substrates, energy of vapored material, deposited material, penetration of deposited material inside other layers (in cases of high energies of vapored particles) which have to be solved by varying the process parameters. This method is convenient, universal, and efficient, as it slightly depends on random factors (for example, human factor); it is easy to control the thickness of the layers; there is a possibility to deposit material on tens of substrates simultaneously, that’s why it is often used in industry. SEM, AFM images, photoluminescence spectra of obtained FAPbBr${}_{2}$I film and electroluminescence spectra of the device are available in Supplementary Information (SI).

### 2.2. Numerical

Transport of carriers is modeled by using the drift-diffusion model which couples the continuity and Poisson equations:

(1) $$\begin{array}{c} \nabla \cdot j_{n}=\nabla \cdot \left(\mu_{n}n\nabla \phi_{n}\right)=-R,\\ \nabla \cdot j_{p}=\nabla \cdot \left(\mu_{p}p\nabla \phi_{p}\right)=R,\\ \nabla \cdot (\varepsilon \nabla \varphi -P)=\rho . \end{array}$$

Here, the first two equations are the continuity equations for electron and hole currents, where *n* and *p* are the electron and hole densities, $\mu_{n}$ and $\mu_{p}$ electron and hole mobility, $\phi_{n}$ and $\phi_{p}$ electron and hole quasi-Fermi levels and *R* recombination rates, respectively. Concerning recombination, we included Shockley-Reed-Hall and direct recombination, which are given by

(2) $$\begin{array}{c} R_{SRH}=\frac{np-n_{i}^{2}}{\tau_{n}\left(p+p_{i}\right)+\tau_{p}\left(n+n_{i}\right)}\\ R_{DIR}=k_{2}\left(np-n_{i}^{2}\right). \end{array}$$

In the Poisson equation—the last equation in Equation (1)—φ is the potential and ρ the total density including free carrier densities, trap distributions, ionized donors, and acceptors. Finally, ε is the permittivity of the material and *P* is the polarization field.

The gold NPs were mimicked by a zero-gap semiconductor, with suitably adjusted band edge energies to reproduce the energy difference between the band edge of the SiO${}_{2}$ and the metal work function, given by the quasi-Fermi level in the gold NP. We heavily doped the valence band with acceptors to create with an excess of carriers a filling situation of electrons in the conduction band, as happens in some metals. In this way, the metallic behavior is reproduced.

Equation (1) are solved with finite elements method (FEM) by TiberCAD simulation tool successfully employed in similar studies [28,29,30]. Details for this model can be found in Refs. [31,32]. Our model is 2D, but the models for 2D and 3D cases give the same results according to our tests.

**Figure 1:**
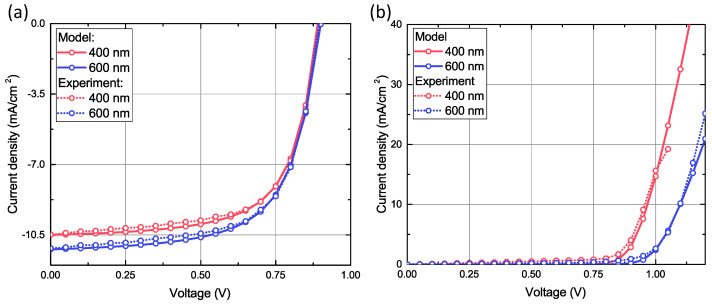
Experimental and modeled I–V characteristics of dual-functional devices with two different thicknesses of perovskite layer: (**a**) 400 nm and (**b**) 600 nm.

We measured two separate series of perovskite devices with different thicknesses of perovskite layers FAPbBr${}_{2}$I: 400 and 600 nm. I–V characteristics of the devices in solar cell regimes were measured under the AM1.5G irradiation (100 mW/cm${}^{2}$). The drift-diffusion modeling was performed with commercial package TiberCAD while the optical generation rate was separately calculated with a Python code based on the Beer–Lambert–Bouguer law. We used initial material parameters provided by the manufacturer and from the literature [33,34,35,36]. After a complex parameter fitting, our numerical model describes well the I–V curves of both devices in two regimes–under the sunlight and in the dark condition. Comparison of numerical and experimental I–V curves for two different thicknesses in solar cell regime is shown in Figure 1. In our measurements, we applied a forward bias voltage. In the solar cell regime at short circuit condition, the generated current flows in the opposite direction. In this sense, the sign of this current is negative. Hence, a negative current under the sunlight means a light-induced current leading to power generation. The device with 600 nm perovskite absorbs more light and, therefore, the short-circuit current is greater in comparison with the device with 400-nm perovskite layer. However, in the LED regime series resistance of this device is larger, which decreases the slope of the open diode region. Based on these results, one can say with confidence that the model with undirectly fitted parameters accurately describes the main physical processes in devices and can be used for further research. Validated material parameters for this LESC are summarized in Table 1.

## 3. Results and Discussion

We consider the integration of a core-shell NP with Au core and SiO${}_{2}$ shell inside the active layer of PeLED. The metal core is completely isolated by a SiO${}_{2}$ shell, thereby preventing chemical degradation of perovskite. We compared this case with pure dielectric NPs and realized that only the situation with a core of gold and a shell of SiO${}_{2}$ can have a significant effect on I–V curves and band diagrams. I–V curve shows that for the structure with Au-SiO${}_{2}$ NPs we can potentially achieve lower currents and switch-on voltage increasing the power efficiency of the device.

**Figure 2:**
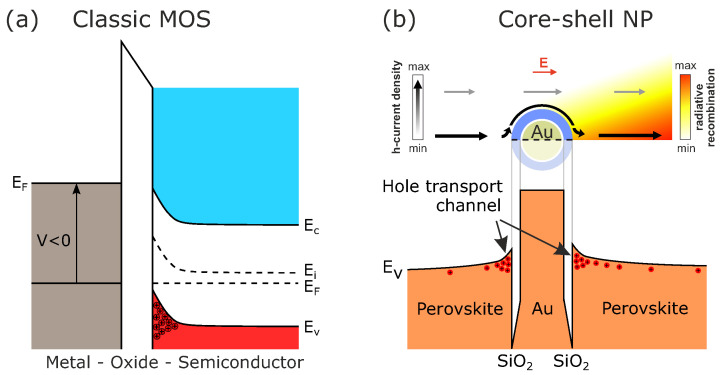
(**a**) Band bending at the interface of insulator/semiconductor in a classic metal-oxide-semiconductor (MOS) structure. (**b**) Valence band profile with formation of hole transport channel around the surface of NP which leads to the increased radiative recombination rate in perovskite.

In the case with a thin layer of SiO${}_{2}$ over the Au core, we observed nano-MOS structure shown in Figure 2. SiO${}_{2}$ as a dielectric material creates the barrier for carriers around the metal (Au), and there is a bending of the conduction and the valence bands. Band structure shows that a hole transport channel forms around this nanoparticle on the external surface of SiO${}_{2}$, which pulls charges coming from the electrode. The effect is even higher for thinner shells and larger core diameters. According to the results of our preliminary simulations, this may lead to the increased radiative recombination rate in perovskite.

We performed the optimization of NP’s morphology: we varied core diameter, shell thickness and location of NP inside the active layer for maximizing the radiative recombination rate. Here we consider the integration of NPs in realistic LESC device described in Section 2.

**Figure 3:**
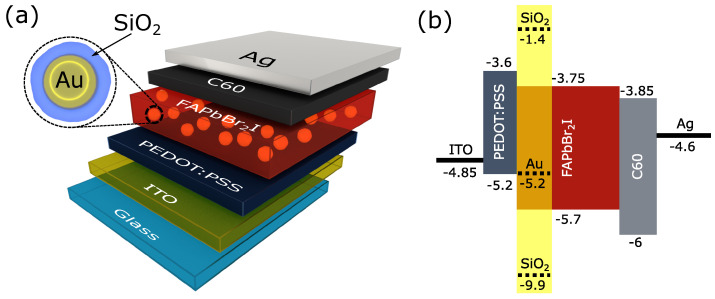
(**a**) Perovskite light-emitting diode (PeLED) structure representation; (**b**) Simplified energy band alignment of the multilayered PeLED device showing the conduction and valence band energy levels with respect to the vacuum level. Yellow insert corresponds to core-shell NPs located inside FAPbBr${}_{2}$I.

Our example corresponds to LESC based on FAPbBr${}_{2}$I with transport layers. Generally, mixed anion halide perovskites are very interesting for photovoltaics since they have a wide bandgap (around 2 eV) and still can absorb a significant part of the sunlight. On the other hand, photoluminescence (and therefore, electroluminescence) can be around 630 nm that corresponds to red color emission. In this way, it is possible to implement a dual-functional device based on this perovskite which is capable to work in two regimes: LED (in red region) and SC. The structure is illustrated in Figure 3. We consider the integration of core-shell NPs in this device, in order to show that NPs can significantly increase the radiative recombination rate in the perovskite layer.

For an efficient electric current generation in the solar cell regime of LESC, one needs to separate the generated charges with transport layers to implement p-i-n structure. In FAPbBr${}_{2}$I LESC, we used PEDOT:PSS as a hole transport layer and C${}_{60}$ as an electron transport layer. As a result, device had the architecture: ITO/PEDOT: PSS/FAPbBr${}_{2}$I/C${}_{60}$/LiF/Ag. A thin layer (about 1 nm) of dielectric LiF forms an electron tunnel junction which screens Schottky barrier from metal contact and makes this contact Ohmic [36,37]. For more details on the device architecture, see description in Section 2. Note that here we study device operation only in the LED regime.

**Figure 4:**
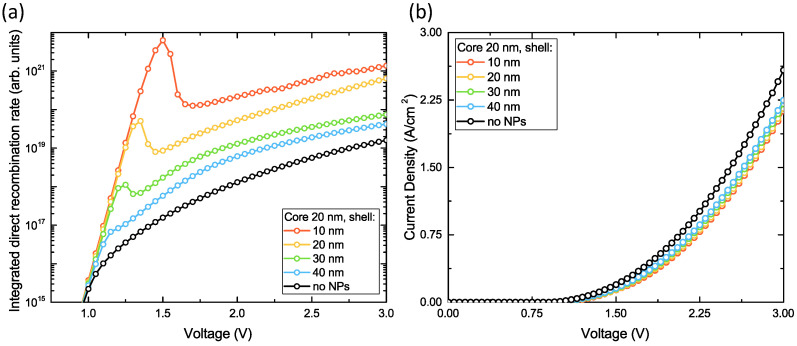
Calculation of optimal shell thickness: (**a**) integrated direct recombination rate for single NP (**b**) I–V curves of the device. Core diameter is fixed and equals 20 nm.

**Figure 5:**
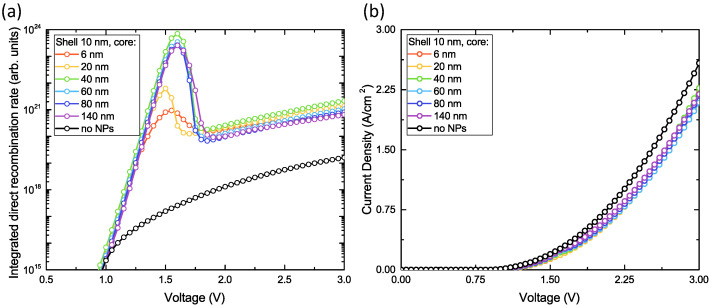
Calculation of optimal core diameter: (**a**) integrated direct recombination rate for single NP (**b**) I–V curves of the device. Shell thickness is fixed and equals 10 nm.

**Figure 6:**
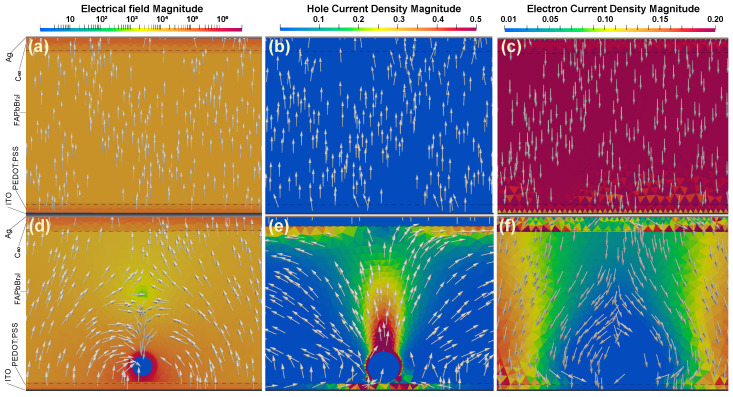
Calculated electrical characteristics of FAPbBr${}_{2}$I at 1.5 V: (**a**) electric field, (**b**) hole current density, (**c**) electric current density distributions, and those with integrated core-shell NP, respectively (**d**–**f**).

In our preliminary simulations, we have found that the optimal location of core-shell NPs in the active layer in terms of radiative recombination enhancement is near the interface of PEDOT:PSS/perovskite (see Figure S6 in SI). For the present design, we performed numerical simulations of electrical characteristics for different values of shell thickness and core diameter and compared the results with the case of bare architecture (without any particle). In our calculations, we considered a single NP located far enough from vertical simulation boundaries so that the whole region affected by inclusion is taken into account. In Figure 4 and Figure 5, we show the corresponding recombination rates and current densities as a function of applied voltage for different sets of geometrical parameters arranged as follows: variable core diameter at fixed shell thickness and vice versa, respectively. Here we can make some interesting observations. First of all, there is a pronounced peak in the direct recombination rate plot at around 1.5 V (for 20 nm core and 10 nm shell). This peak vanishes with the increase of insulator thickness and shifts towards lower voltage values. On the contrary, the effect can be further enhanced if we increase the diameter of the metal core until a certain threshold after which the operation of the device starts to degrade. So the maximal performance is achieved at 40 nm gold core diameter and 10 nm insulating shell. The second noticeable phenomenon is related to I–V curves where the current densities are always lower for designs with NP as compared to bare structures at the same voltage applied. That means higher power efficiency which is inversely proportional to the operational voltage. To explain this quite interesting behavior, we calculated field and current distributions at 1.5 V shown in Figure 6.

Unlike in classic MOS-type devices, the metal core of our NP is not directly connected to any external bias which would allow to modulate carrier concentration being fully isolated by a silica shell. However, it is affected by the potential difference across the device when we apply voltage since NP is located inside the perovskite layer. In such way, the electric field turns on the MOS functionality of core-shell NP which results in the formation of a transport channel for holes due to favorable bending of valence band at its surface. Holes move around the perovskite/SiO${}_{2}$ interface and then start to spread from the region above particle towards C${}_{60}$ layer. As a result, holes accelerated through the formed channel accumulate along the perovskite/C${}_{60}$ interface. Here they meet the energy barrier formed by C${}_{60}$ which prevents their further penetration. In Figure 6 one can see the regions where the current density for holes is higher as compared to the bare case. Gray arrows in Figure 6 are streamlines that correspond to the direction of propagation. On the contrary, current density for electrons is suppressed around the particle and the reason for this is the formation of a region acting as recombination center near perovskite/C${}_{60}$ interface at 1.5 V. At the range of 1.5–1.75 V, the efficiency of transport channel for holes decreases and the radiative recombination rate has a negative trend. At higher voltages (>1.75 V), the trend of the radiative recombination rate is reversed again and gradually increases with the voltage being still higher as compared to the bare case. We calculated IQE (available in SI) and found that our LESC can reach 2% at 1.5 V with optimal geometry of NP that is $2\times 10^{5}$ times larger than the highest IQE for the bare case achieved at 3 V. If one compare both cases at the same voltage (3 V), NP still grants a hundred times enhanced IQE. Additionally, we simulated the situation of three NPs located in the modeling box (Figures S7 and S8 of SI). The results confirm that the chosen particle concentration is small enough for considering NPs isolated from each other.

**Figure 7:**
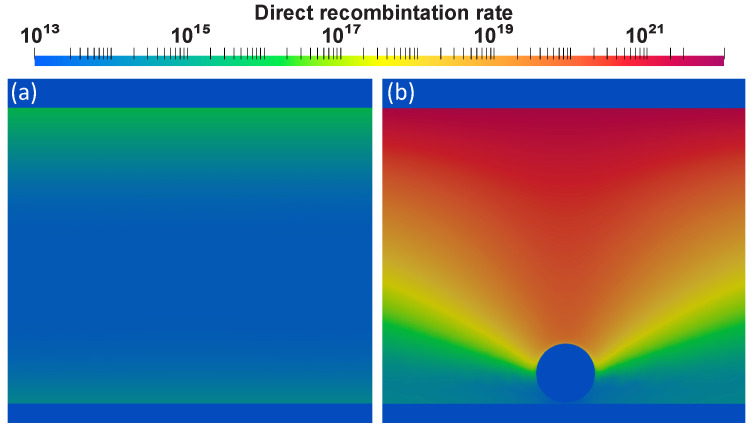
(**a**) Direct recombination in FAPbBr${}_{2}$I device at 1.5V without nanoparticles (NPs) and (**b**) with core-shell NP of the following geometry: 60 nm diameter of gold core and 10 nm thickness of SiO${}_{2}$ shell.

In the direct recombination distribution (Figure 7), we observe maximal rate above the particles as compared to the bare structure. Such hot spots are controlled by contact properties and particle location. As we have arranged the contacts in the structure, we have the flow of electrons coming from the top of the structure, while the holes come from the bottom. Now, while the holes tend to move through the channel formed by NP, the electrons, coming from the upper part, recombine mainly with the first holes they encounter in their path, namely those in the upper part of the NP and near the perovskite/C${}_{60}$ interface.

**Figure 8:**
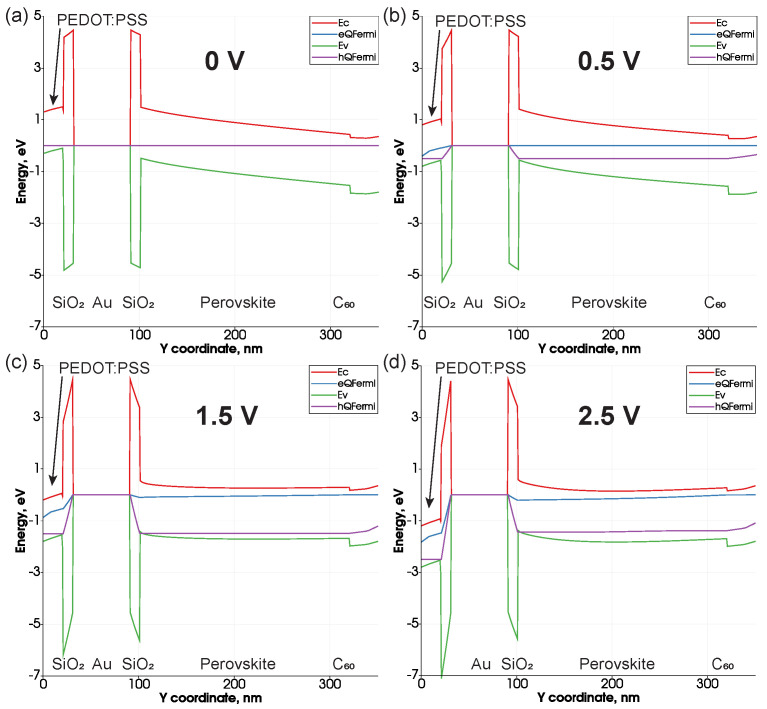
Energy band diagrams along the growth direction of the structure for FAPbBr${}_{2}$I device with integrated NP (60 nm diameter of gold core and 10 nm thickness of SiO${}_{2}$ shell) at four different voltages: (**a**) 0 V (**b**) 0.5 V (**c**) 1.5 V (**d**) 2.5 V.

To prove the presence of hole transport channel, we investigated the energy band diagrams across the structure at different voltages (0 V, 0.5 V, 1.5 V and 2.5 V). When the bands are in equilibrium (Figure 8a), no charge transfer occurs. As the bias increases, the electron and hole Fermi levels approach each other, which leads to the appearance of a current. In our calculations of integrated direct recombination, we observed a special case at bias around 1.5 V. In Figure 8c, the reason for this phenomena can be seen, hole Fermi level starts to cross the valence band on the boundary between perovskite and SiO${}_{2}$, which leads to the formation of a typical band diagram for MOS structure and, therefore, accumulation of holes in this area. Also, the maximum convergence of the valence band and hole Fermi level is observed. With a further increase in the voltage, the intersection of the zones around the nanoparticle remains. However, in the perovskite volume, they begin to move away from each other slightly (Figure 8d), which leads to a decrease in the recombination rate.

## 4. Conclusions

In this work, we demonstrate a novel approach for PeLED efficiency enhancement by band structure engineering with metal-dielectric NPs. We show that Au/SiO${}_{2}$ NPs integrated into a red FAPbBr${}_{2}$I-based dual-functional device leads to an increase of the radiative recombination rate by four orders of magnitude due to the formation of MOS interfaces. This superior performance is achieved by NPs with optimal Au core diameter of 40 nm and SiO${}_{2}$ shell thickness of 10 nm located near the PEDOT:PSS/perovskite interface. Compared to the bare case, these NPs allow lower turn-on voltage and enable up to $2\times 10^{5}$ times larger IQE. This method of efficiency enhancement has considerable potential for PeLEDs of various types.

## Figures and Tables

**Table 1:** Verified materials parameters of light-emitting solar cell (LESC) used in electrical simulations.

Parameter	PEDOT:PSS	FAPbBr${}_{2}$I	C${}_{60}$	Unit
Thickness, d	10	400/600	30	nm
Valence (HOMO) Energy, E_v_	−5.2	−5.7	−6	eV
Bandgap Energy, E_g_	1.6	1.95	2.15	eV
Hole Mobility, $\mu_{p}$	1.1	2	1.6	cm${}^{2}$/(V·s)
Electron Mobility, $\mu_{e}$	0.45	2	0.1	cm${}^{2}$/(V·s)
Doping concentration	10${}^{17}$	-	10${}^{17}$	cm${}^{-3}$
SRH recombination (bulk/surface), $\tau_{SRH}$	10${}^{-6}$	2× 10${}^{-8}$/10${}^{-10}$	10${}^{-6}$	s
Direct recombination (bulk/surface), $\tau_{dir}$	–	2 × 10${}^{-8}$/2 × 10${}^{-6}$	–	s
ITO work function	−4.85	–	–	eV
Ag work function	–	–	−4.6	eV

## Data Availability

Data is contained within the article or supplementary material.

## Supplementary Materials

The following are available online at https://www.mdpi.com/2079-4991/11/1/45/s1, Figure S1: SEM image FAPbBr${}_{2}$I film, Figure S2: AFM image of FAPbBr${}_{2}$I film, Figure S3: Measured electroluminescence spectra of FAPbBr${}_{2}$I device at 2V, Figure S4: Calculated IQE characteristics for optimized architectures, Figure S5: Measured photoluminescence spectra of FAPbBr${}_{2}$I film, Figure S6: Radiative recombination rate for different core-shell nanoparticle location at 1.5 V, Figure S7: Electric field, electron and hole current density distributions for three nanoparticles, Figure S8: Radiative recombination rate for three nanoparticles.

## Abbreviations

The following abbreviations are used in this manuscript:

PeLED	perovskite light-emitting diode
LESC	light-emitting solar cell
MOS	metal-oxide-semiuconductor
SC	solar cell
EQE	external quantum efficiency
IQE	internal quantum efficiency
NP	nanoparticle
