# Supplementary material for: Giant Enhancement of Radiative Recombination in Perovskite Light-Emitting Diodes with Plasmonic Core-Shell Nanoparticles
Source: Nanomaterials (Basel). 2020 Dec 27;11(1):45. doi: 10.3390/nano11010045 (PMC7823440; doi:10.3390/nano11010045)
Supplement: Supplementary file 1 [file nanomaterials-11-00045-s001.pdf]

Supplementary Materials: Giant Enhancement of Radiative Recombination in Perovskite Light-emitting Diodes with Plasmonic Core-shell Nanoparticles

Mikhail A. Masharin [1,‡], Alexander S. Berestennikov [1,3‡], Daniele Barettin [2], Pavel M. Voroshilov [1*], Konstantin S. Ladutenko [1], Aldo Di Carlo [3], and Sergey V. Makarov [1]

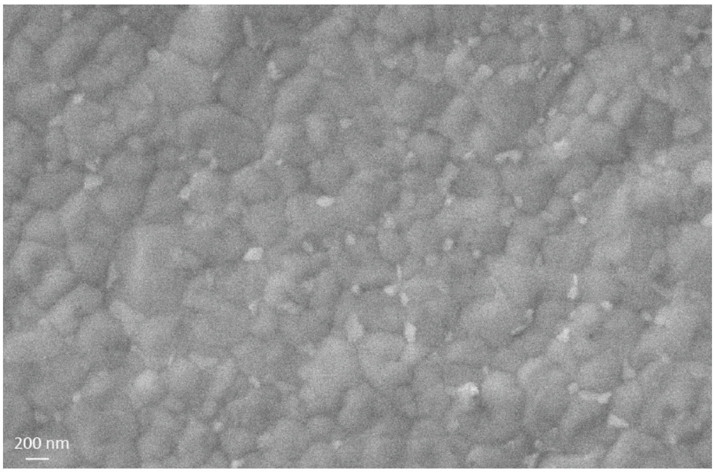

Figure S1. SEM image $FAPbBr_2I$ film.

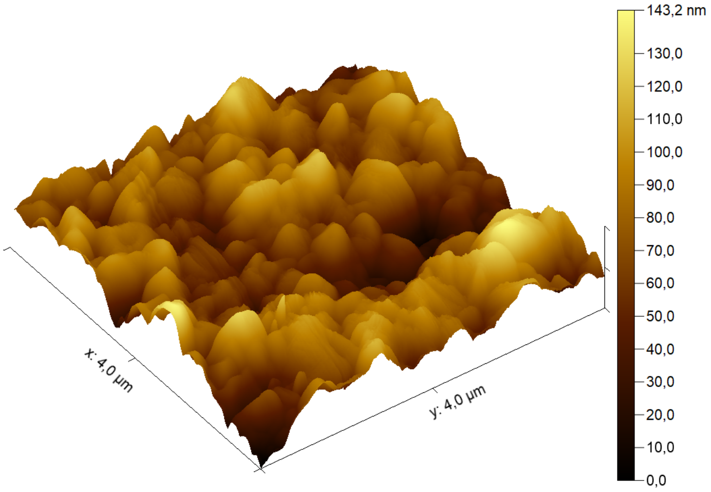

Figure S2. AFM image of $FAPbBr_2I$ film.

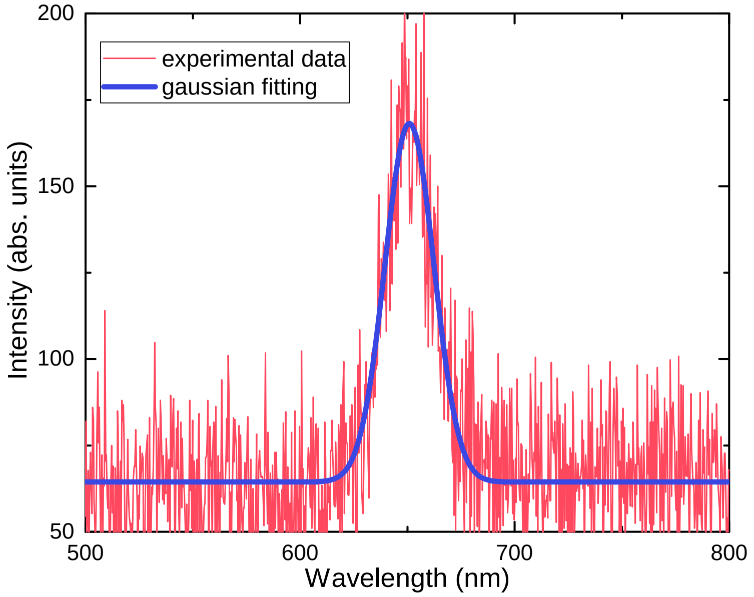

Figure S3. Measured electroluminescence spectra of $FAPbBr_2I$ device at 2V.

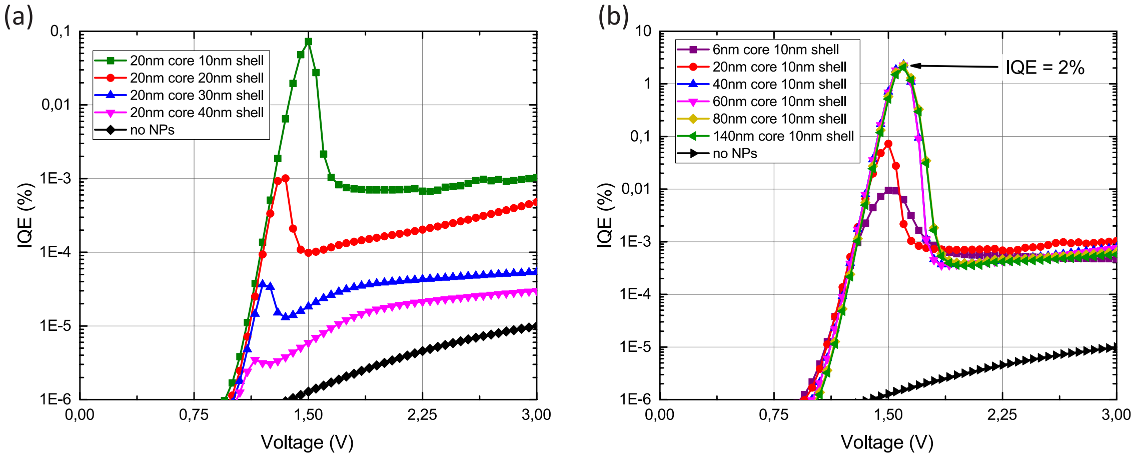

Figure S4. Calculated IQE characteristics for optimized architectures.

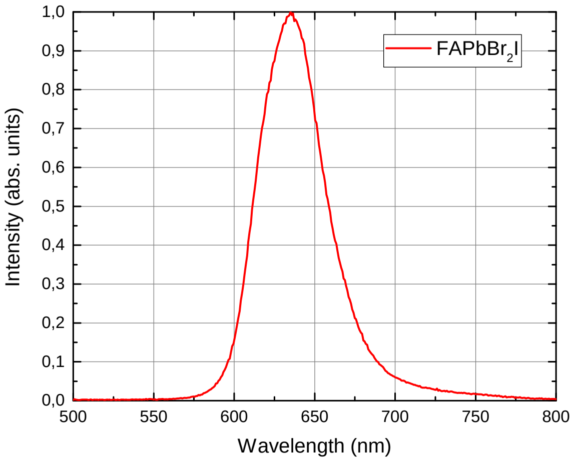

Figure S5. Measured photoluminescence spectra of $FAPbBr_2I$ film.

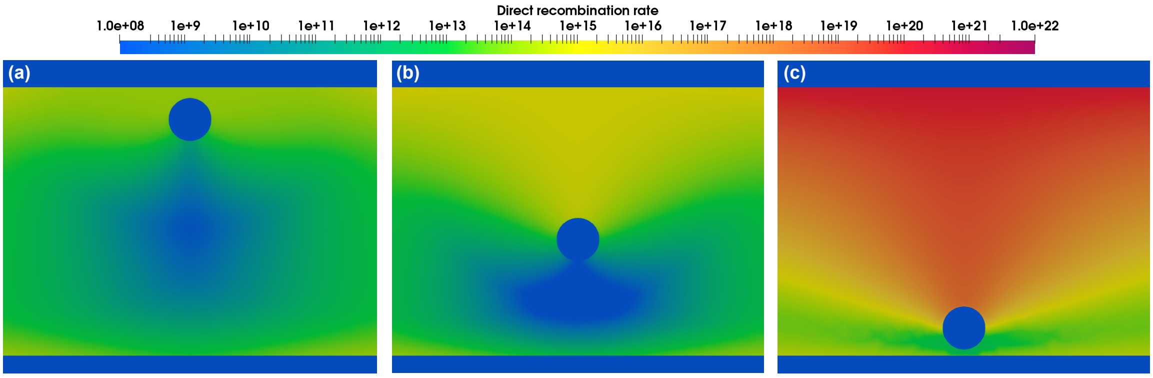

Figure S6. Radiative recombination rate for different core-shell nanoparticle location at 1.5V.

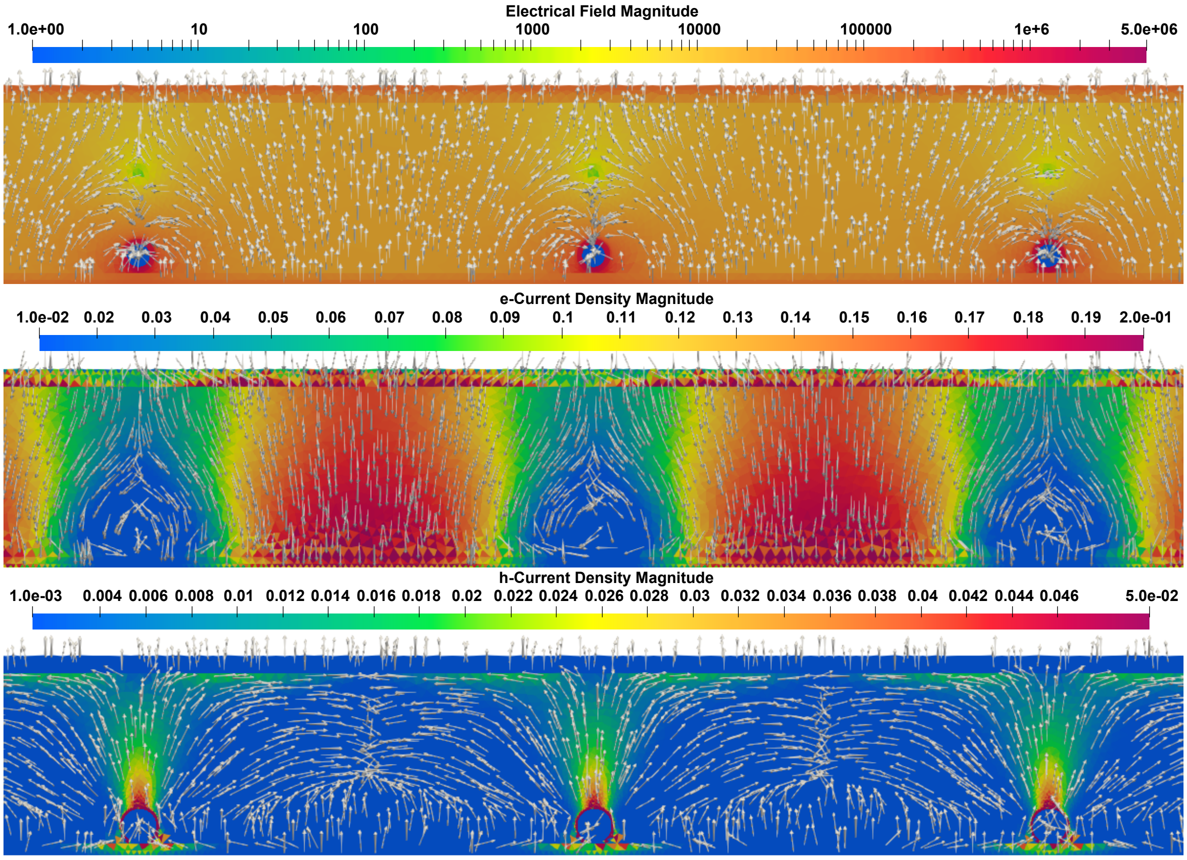

Figure S7. Electric field, electron and hole current density distributions for three nanoparticles.

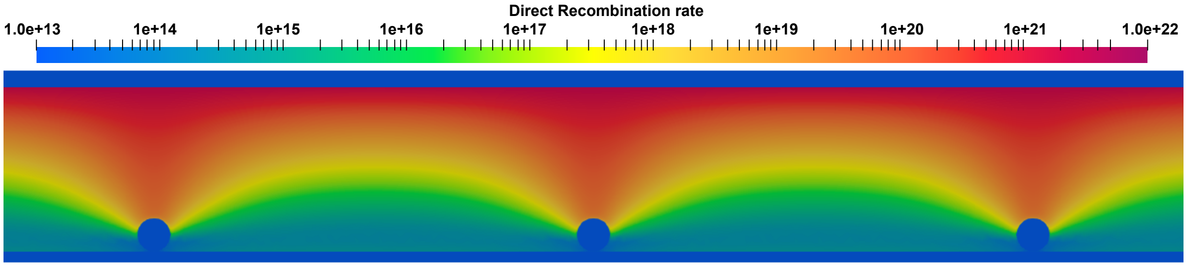

Figure S8. Radiative recombination rate for three nanoparticles.
